# Supplementary material for: Selection of reference genes for quantitative analysis of microRNA expression in three different types of cancer
Source: PLoS One. 2022 Feb 17;17(2):e0254304. doi: 10.1371/journal.pone.0254304 (PMC8853544; doi:10.1371/journal.pone.0254304)
Supplement: S1 Table — (DOCX) [file pone.0254304.s001.docx]

Table 1. Patient characteristics at the time of diagnosis (thyroid tissue).

| Factor | Value |
| --- | --- |
| Gender |  |
| Male | 21 |
| Female | 11 |
| Age (years) |  |
| >60 | 7 |
| <60 | 25 |
| normal thyroid tissue | 16 |
| hyperplastic nodule | 5 |
| papillary thyroid carcinoma | 2 |
| follicular variant of papillary thyroid carcinoma | 2 |
| follicular thyroid adenoma | 6 |
| follicular thyroid carcinoma | 1 |
