# Supplementary material for: Selection of reference genes for quantitative analysis of microRNA expression in three different types of cancer
Source: PLoS One. 2022 Feb 17;17(2):e0254304. doi: 10.1371/journal.pone.0254304 (PMC8853544; doi:10.1371/journal.pone.0254304)
Supplement: S2 Table — (DOCX) [file pone.0254304.s002.docx]

Table 2. Patient characteristics at the time of diagnosis (brain tissue).

| Factor | Value |
| --- | --- |
| Gender |  |
| Male | 3 |
| Female | 3 |
| Age (years) |  |
| >60 | 2 |
| <60 | 4 |
| Grade |  |
| I | 1 |
| II | 2 |
| III | 2 |
| IV | 1 |
