# Supplementary material for: Selection of reference genes for quantitative analysis of microRNA expression in three different types of cancer
Source: PLoS One. 2022 Feb 17;17(2):e0254304. doi: 10.1371/journal.pone.0254304 (PMC8853544; doi:10.1371/journal.pone.0254304)
Supplement: S3 Table — (DOCX) [file pone.0254304.s003.docx]

Table 3. Patient characteristics at the time of diagnosis (bone marrow tissue).

| Factor | Value |
| --- | --- |
| **non-Hodgkin’s lymphomas (n=6)** | |
| DLBCL | 6 |
| Gender |  |
| Male | 4 |
| Female | 2 |
| Age (years) |  |
| >60 | 3 |
| <60 | 3 |
| LDH |  |
| Increased | 4 |
| Normal | 2 |
| Clinical stage |  |
| I+II | 3 |
| III+IV | 3 |
| IPI score |  |
| High‑risk | 6 |
| median high‑risk |  |
| Low‑risk |  |
| Myelodysplasia |  |
| Yes | 3 |
| No | 3 |
| Anemia |  |
| Yes | 3 |
| No | 3 |
| **Myelodysplastic syndromes (n=3)** | |
| Gender |  |
| Male | 0 |
| Female | 3 |
| Age (years) |  |
| >60 | 1 |
| <60 | 2 |
| Hemoglobin, g/L | 78(72-91) |
| WBC count, × 10^3^/L | 4.7(4-6.25) |
| ANC, /dL | 4.1(2,9-5.3) |
| Circulating blast count, % | 0(0-4) |
| Platelet count, × 10^3^/L | 145(97.5-182) |
| LDH (conversion), mg/L | 196(128.25-380) |
| Bone marrow cellularity, % | 71(55-75.25) |
| Bone marrow blasts, % | <5 |
| MDS subtype |  |
| RCUD | 1 |
| RAEB | 1 |
| RCMD | 1 |
| Cytogenetic risk group |  |
| Good |  |
| Normal | 1 |
| Intermediate | 2 |
| Poor |  |
| R-IPSS risk category |  |
| Very low | 1 |
| Low |  |
| Intermediat | 1 |
| High |  |
| Very high | 1 |
| **non-cancerous blood diseases (n=3)** | |
| Gender |  |
| Male | 1 |
| Female | 2 |
| Age (years) |  |
| >60 | 1 |
| <60 | 2 |
| Hemoglobin, g/L | 92(76.25-110) |
| WBC count, × 10^3^/L | 6.9(4.7-9.1) |
| ANC, /dL | 5.2(3.2-7) |
| Platelet count, × 10^3^/L | 220(125.75-280) |
| Iron-deficiency anemia | 1 |
| B12 deficiency anemia | 1 |
| immune thrombocytopenia | 1 |

Values are presented as number (%) or median (range).

DLBCL, diffuse large B cell lymphoma; LDH, lactate dehydrogenase; IPI, International Prognostic Index.

MDS, myelodysplastic syndrome; RCUD, refractory cytopenia with unilineage dysplasia; RCMD, refractory cytopenia with multilineage dysplasia; RAEB, refractory anemia with excess of blasts; R-IPSS, revised international prognostic scoring system .
