# Supplementary material for: Selection of reference genes for quantitative analysis of microRNA expression in three different types of cancer
Source: PLoS One. 2022 Feb 17;17(2):e0254304. doi: 10.1371/journal.pone.0254304 (PMC8853544; doi:10.1371/journal.pone.0254304)
Supplement: S4 Table — The names of the microRNA’s together with the observed values of deviations are reported. (DOCX) [file pone.0254304.s004.docx]

**Table 4.** MicroRNAs with the least variable expression in all groups of patients. The names of the microRNA’s together with the observed values of deviations are reported.

| Normalization / norm | 1 | 2 | 3 | 4 | 5 | 6 | 7 | 8 | 9 | 10 |
| --- | --- | --- | --- | --- | --- | --- | --- | --- | --- | --- |
| Normalization to housekeeping genes | | | | | | | | | | |
| SD | miR-376a-3p | miR-532-5p | miR-362-5p | miR-302d-3p | miR-423-5p | miR-590-5p | miR-192-5p | miR-10a-5p | miR-28-5p | miR-320e |
|  | 1.62 | 1.65 | 1.65 | 1.66 | 1.77 | 1.77 | 1.78 | 1.78 | 1.83 | 1.87 |
| Range | miR-376a-3p | miR-10a-5p | miR-320e | miR-590-5p | miR-151a-5p | miR-423-3p | miR-532-5p | miR-331-3p | let-7b-5p | miR-192-5p |
|  | 7.47 | 8.21 | 8.36 | 8.41 | 8.53 | 8.65 | 8.67 | 8.77 | 8.78 | 8.79 |
| IQR | miR-362-5p | miR-425-5p | miR-191-5p | miR-28-5p | miR-361-3p | miR-192-5p | let-7d-5p | miR-10a-5p | miR-423-5p | miR-302d-3p |
|  | 1.50 | 1.63 | 1.68 | 1.75 | 1.76 | 1.83 | 1.85 | 2.00 | 2.02 | 2.08 |
| MADM | miR-362-5p | miR-532-5p | miR-302d-3p | miR-376a-3p | miR-192-5p | miR-10a-5p | miR-423-5p | miR-28-5p | miR-191-5p | miR-590-5p |
|  | 1.17 | 1.26 | 1.29 | 1.29 | 1.33 | 1.33 | 1.33 | 1.35 | 1.36 | 1.36 |
| Normalization to total miRNA content | | | | | | | | | | |
| SD | miR-425-5p | miR-106b-5p | miR-362-5p | let-7d-5p | miR-423-5p | let-7i-5p | miR-140-5p | miR-191-5p | miR-28-5p | miR-19a-3p |
|  | 0.63 | 0.77 | 0.79 | 0.79 | 0.79 | 0.82 | 0.86 | 0.87 | 0.90 | 0.91 |
| Range | miR-362-5p | miR-106b-5p | miR-425-5p | miR-140-5p | let-7d-5p | miR-423-5p | miR-191-5p | miR-28-5p | miR-93-5p | miR-19a-3p |
|  | 3.13 | 3.28 | 3.32 | 3.51 | 3.56 | 3.77 | 3.78 | 3.94 | 4.14 | 4.50 |
| IQR | miR-99b-5p | miR-151a-3p | miR-361-3p | let-7i-5p | miR-296-5p | let-7a-5p | miR-28-5p | miR-423-5p | miR-106b-5p | miR-532-5p |
|  | 0.83 | 0.86 | 0.88 | 0.90 | 0.92 | 0.93 | 0.93 | 0.95 | 0.96 | 0.96 |
| MADM | miR-425-5p | miR-106b-5p | let-7i-5p | miR-423-5p | let-7d-5p | miR-362-5p | let-7a-5p | miR-28-5p | miR-140-5p | miR-532-5p |
|  | 0.50 | 0.58 | 0.62 | 0.63 | 0.64 | 0.65 | 0.65 | 0.66 | 0.67 | 0.68 |
| Normalization to 75 highest expressed miRNA content | | | | | | | | | | |
| SD | miR-425-5p | let-7i-5p | let-7d-5p | miR-652-3p | miR-106b-5p | miR-191-5p | miR-423-5p | miR-93-5p | miR-362-5p | miR-140-5p |
|  | 0.69 | 0.76 | 0.78 | 0.83 | 0.85 | 0.86 | 0.86 | 0.87 | 0.93 | 0.94 |
| Range | miR-423-5p | miR-191-5p | miR-93-5p | miR-425-5p | let-7i-5p | let-7d-5p | miR-140-5p | miR-652-3p | miR-362-5p | miR-106b-5p |
|  | 3.32 | 3.41 | 3.42 | 3.48 | 3.79 | 3.91 | 4.14 | 4.19 | 4.28 | 4.46 |
| IQR | let-7a-5p | miR-425-5p | miR-361-3p | miR-151a-3p | miR-28-5p | miR-361-5p | miR-532-5p | miR-423-5p | miR-106b-5p | let-7i-5p |
|  | 0.74 | 0.76 | 0.86 | 0.92 | 0.95 | 0.98 | 0.98 | 1.00 | 1.01 | 1.03 |
| MADM | miR-425-5p | let-7i-5p | let-7d-5p | miR-652-3p | miR-106b-5p | miR-93-5p | miR-19a-3p | let-7a-5p | miR-423-5p | miR-191-5p |
|  | 0.53 | 0.60 | 0.62 | 0.63 | 0.64 | 0.66 | 0.69 | 0.70 | 0.70 | 0.73 |
| Normalization to positive controls | | | | | | | | | | |
| SD | miR-302d-3p | miR-376a-3p | miR-10a-5p | miR-423-5p | miR-590-5p | miR-362-5p | let-7b-5p | miR-28-3p | miR-423-3p | miR-425-5p |
|  | 1.23 | 1.30 | 1.39 | 1.42 | 1.44 | 1.45 | 1.52 | 1.54 | 1.59 | 1.61 |
| Range | miR-10a-5p | miR-376a-3p | miR-590-5p | miR-423-3p | miR-302d-3p | miR-151a-3p | miR-28-3p | miR-331-3p | miR-4516 | let-7b-5p |
|  | 5.87 | 5.95 | 6.02 | 6.38 | 6.50 | 6.56 | 6.63 | 6.69 | 6.71 | 6.77 |
| IQR | miR-362-5p | miR-302d-3p | miR-425-5p | miR-93-5p | let-7i-5p | miR-10a-5p | miR-423-5p | miR-191-5p | miR-15a-5p | let-7d-5p |
|  | 1.10 | 1.44 | 1.46 | 1.52 | 1.62 | 1.63 | 1.72 | 1.73 | 1.77 | 1.81 |
| MADM | miR-362-5p | miR-302d-3p | miR-376a-3p | miR-423-5p | miR-425-5p | miR-10a-5p | miR-191-5p | let-7d-5p | miR-28-5p | miR-28-3p |
|  | 0.90 | 0.91 | 1.04 | 1.06 | 1.07 | 1.08 | 1.18 | 1.20 | 1.20 | 1.20 |

**Abbreviations:** SD = standard deviation, IQR = inter-quartile range, MADM = mean absolute deviation from the median.
