# Supplementary material for: Selection of reference genes for quantitative analysis of microRNA expression in three different types of cancer
Source: PLoS One. 2022 Feb 17;17(2):e0254304. doi: 10.1371/journal.pone.0254304 (PMC8853544; doi:10.1371/journal.pone.0254304)
Supplement: S7 Table — The names of the miRNAs together with the observed values of deviations are reported. If the names of two miRNAs are linked with the plus sign “+”, then only the total content of these two miRNAs was measured. (DOCX) [file pone.0254304.s007.docx]

**Table 7.** MicroRNAs with the least variable expression in brain tissue. The names of the miRNAs together with the observed values of deviations are reported. If the names of two miRNAs are linked with the plus sign “+”, then only the total content of these two miRNAs was measured.

| Normalization / norm | 1 | 2 | 3 | 4 | 5 | 6 | 7 | 8 | 9 | 10 |
| --- | --- | --- | --- | --- | --- | --- | --- | --- | --- | --- |
| Normalization to housekeeping genes | | | | | | | | | | |
| SD | miR-505-3p | miR-24-3p | miR-374a-5p | miR-28-5p | miR-99b-5p | miR-140-5p | miR-1915-3p | miR-151a-3p | miR-4508 | miR-454-3p |
|  | 0.49 | 0.60 | 0.60 | 0.61 | 0.62 | 0.62 | 0.64 | 0.66 | 0.66 | 0.66 |
| Range | miR-505-3p | miR-1915-3p | miR-24-3p | miR-99b-5p | miR-140-5p | miR-590-5p | miR-151a-3p | miR-374a-5p | miR-19b-3p | miR-454-3p |
|  | 1.32 | 1.79 | 1.94 | 1.95 | 1.95 | 1.96 | 2.03 | 2.04 | 2.09 | 2.19 |
| IQR | miR-423-5p | miR-503 | let-7b-5p | miR-19a-3p | miR-320e | miR-181c-5p | miR-4488 | miR-106a-5p + miR-17-5p | miR-28-5p | miR-20a-5p + miR-20b-5p |
|  | 0.44 | 0.61 | 0.62 | 0.62 | 0.69 | 0.72 | 0.72 | 0.73 | 0.73 | 0.73 |
| MADM | miR-505-3p | miR-374a-5p | miR-28-5p | miR-19a-3p | miR-24-3p | miR-186-5p | miR-99b-5p | miR-140-5p | miR-106a-5p + miR-17-5p | miR-4508 |
|  | 0.41 | 0.43 | 0.45 | 0.45 | 0.47 | 0.49 | 0.49 | 0.49 | 0.50 | 0.50 |
| Normalization to total miRNA content | | | | | | | | | | |
| SD | miR-98 | miR-500a-5p + miR-501-5p | miR-361-5p | let-7d-5p | miR-15a-5p | miR-148b-3p | let-7a-5p | miR-660-5p | miR-194-5p | let-7g-5p |
|  | 0.31 | 0.34 | 0.35 | 0.35 | 0.36 | 0.38 | 0.39 | 0.39 | 0.39 | 0.40 |
| Range | miR-98 | miR-30e-5p | miR-30d-5p | miR-148b-3p | miR-484 | miR-500a-5p + miR-501-5p | miR-15a-5p | let-7d-5p | miR-146a-5p | miR-888-5p |
|  | 0.94 | 1.04 | 1.12 | 1.13 | 1.17 | 1.17 | 1.18 | 1.27 | 1.28 | 1.28 |
| IQR | let-7a-5p | miR-331-3p | let-7d-5p | miR-532-5p | miR-185-5p | miR-489 | miR-191-5p | let-7b-5p | miR-194-5p | miR-494 |
|  | 0.23 | 0.26 | 0.39 | 0.41 | 0.41 | 0.43 | 0.44 | 0.44 | 0.45 | 0.45 |
| MADM | let-7a-5p | let-7d-5p | miR-361-5p | miR-98 | miR-500a-5p + miR-501-5p | miR-532-5p | miR-194-5p | miR-148b-3p | let-7g-5p | miR-15a-5p |
|  | 0.22 | 0.25 | 0.25 | 0.26 | 0.27 | 0.27 | 0.27 | 0.27 | 0.29 | 0.29 |
| Normalization to 75 highest expressed miRNA content | | | | | | | | | | |
| SD | miR-98 | miR-15a-5p | let-7d-5p | miR-500a-5p + miR-501-5p | miR-361-5p | miR-194-5p | miR-148b-3p | let-7a-5p | miR-660-5p | let-7g-5p |
|  | 0.32 | 0.34 | 0.36 | 0.36 | 0.37 | 0.37 | 0.39 | 0.40 | 0.40 | 0.41 |
| Range | miR-98 | miR-15a-5p | miR-30e-5p | miR-30d-5p | miR-484 | miR-148b-3p | miR-500a-5p + miR-501-5p | let-7d-5p | miR-146a-5p | miR-888-5p |
|  | 0.98 | 1.11 | 1.13 | 1.15 | 1.19 | 1.19 | 1.23 | 1.27 | 1.27 | 1.28 |
| IQR | let-7a-5p | miR-331-3p | miR-532-5p | miR-185-5p | let-7d-5p | miR-194-5p | miR-489 | miR-361-5p | miR-191-5p | let-7b-5p |
|  | 0.22 | 0.28 | 0.38 | 0.39 | 0.42 | 0.42 | 0.45 | 0.45 | 0.46 | 0.46 |
| MADM | let-7a-5p | let-7d-5p | miR-361-5p | miR-194-5p | miR-98 | miR-532-5p | miR-500a-5p + miR-501-5p | miR-15a-5p | miR-148b-3p | let-7g-5p |
|  | 0.22 | 0.25 | 0.25 | 0.26 | 0.27 | 0.27 | 0.27 | 0.28 | 0.28 | 0.30 |
| Normalization to positive controls | | | | | | | | | | |
| SD | miR-579 | miR-22-3p | miR-378e | miR-361-3p | miR-423-3p | miR-500a-5p + miR-501-5p | miR-148b-3p | miR-320a | miR-10a-5p | miR-28-3p |
|  | 0.24 | 0.45 | 0.48 | 0.48 | 0.51 | 0.51 | 0.52 | 0.52 | 0.53 | 0.54 |
| Range | miR-579 | miR-423-3p | miR-361-3p | miR-10a-5p | miR-320a | miR-500a-5p + miR-501-5p | miR-22-3p | miR-151a-5p | miR-378e | miR-489 |
|  | 0.84 | 1.29 | 1.38 | 1.38 | 1.45 | 1.60 | 1.67 | 1.68 | 1.73 | 1.78 |
| IQR | miR-579 | miR-22-3p | miR-378e | miR-185-5p | let-7b-5p | miR-151a-5p | miR-148b-3p | miR-489 | miR-193b-3p | miR-23a-3p |
|  | 0.37 | 0.41 | 0.47 | 0.64 | 0.65 | 0.67 | 0.69 | 0.70 | 0.70 | 0.71 |
| MADM | miR-579 | miR-22-3p | miR-378e | miR-361-3p | miR-28-3p | miR-148b-3p | miR-489 | let-7b-5p | miR-151a-5p | miR-500a-5p + miR-501-5p |
|  | 0.20 | 0.31 | 0.33 | 0.39 | 0.40 | 0.40 | 0.40 | 0.41 | 0.41 | 0.42 |

**Abbreviations:** SD = standard deviation, IQR = inter-quartile range, MADM = mean absolute deviation from the median.
