# Supplementary material for: Selection of reference genes for quantitative analysis of microRNA expression in three different types of cancer
Source: PLoS One. 2022 Feb 17;17(2):e0254304. doi: 10.1371/journal.pone.0254304 (PMC8853544; doi:10.1371/journal.pone.0254304)
Supplement: Attachment — Submitted filename: Review.docx [file pone.0254304.s008.docx]

The aim of this article was to find the miRNAs with stable expression in the thyroid gland, brain and bone marrow cancer and normal tissues. These miRNAs supposed to be suitable as endogenous controls for miRNAs quantification experiments. RT-qPCR requires endogenous controls for result normalization and reliability. The endogenous control helps to correct differences between sample quality and variations during RNA extraction or reverse transcription procedures. Housekeeping genes, ribosomal, small nuclear or nucleolar RNAs can play the role of such internal controls. However, expression levels of these genes may differ in neoplastic and normal tissues. To find proper endogenous control, the authors evaluated expression stability about of 800 miRNAs with help of NanoString nCounter Assay for miRNAs quantification. Their recommendations are to use as internal controls the following miRNAs: miR-361-3p, -151a-3p and -29b- 3p for thyroid gland; miR-15a-5p, -194-5p and -532-5p for brain; miR-140-5p, -148b- 3p and -362-5p for bone marrow; and miR-423-5p, -28-5p and -532-5p as the universal controls for all three tissue types. I suppose this important paper will be useful for the future investigations in the field of RT-qPCR-based miRNAs quantification. But, I have several questions for the authors:

1. On the page 5, lines 116-117, the authors wrote : “the control group consisted of patients with non-cancerous blood diseases”. What was the reason to use these patients as the control group? Because different diseases, even non-cancerous, can significantly change miRNAs expression profile in patients tissues in compare with healthy ones.
2. Would the authors kindly provide a list of housekeeping genes and positive controls, used for normalization?
3. Let-7a, -7b and -7d were listed in Table 1 among a least variable miRNAs in thyroid gland, brain and as universal controls correspondently. But, miRNAs from let-7 family are well known tumor suppressors. Expression of these miRs is downregulated in tumors. How can it be possible that these miRNAs are the most stable?
4. There is no confidence interval for p-values in the Table 2. Significant p-values in this table may be shown in bold or color.
5. MiR-28-5p and miR-532-5p were chosen like the universal controls for all three tissues. But, among 10 least variable miRNAs, miR-28-5p is only ninth in thyroid gland and even was not listed in Table 1 for brain and bone marrow. The same story is for miR-532-5p. This miRNA is stable in brain tissues only. What is the possible reason for these discrepancies?
