# Supplementary material for: Selection of reference genes for quantitative analysis of microRNA expression in three different types of cancer
Source: PLoS One. 2022 Feb 17;17(2):e0254304. doi: 10.1371/journal.pone.0254304 (PMC8853544; doi:10.1371/journal.pone.0254304)
Supplement: Attachment — Submitted filename: Response to Reviewers.doc [file pone.0254304.s009.doc]

We thank the Reviewers for their insightful comments. Below are our answers to the Reviewers’ comments shown in bold.

Reviewer #1

1. **On the page 5, lines 116-117, the authors wrote: “the control group consisted of patients with non-cancerous blood diseases”. What was the reason to use these patients as the control group? Because different diseases, even non-cancerous, can significantly change miRNAs expression profile in patients’ tissues in compare with healthy ones.**

We have added the following explanation on p.5

“Work with healthy bone marrow donors for allogeneic transplantation is beyond the competence of our clinic. Taken together, we decided to go with a control group composed of people who had no hematologic cancer, but had indications for bone marrow examination to exclude one. They were people with secondary anemic and cytopenic conditions, in whom leukemias were not confirmed by myelography.”

2. **Would the authors kindly provide a list of housekeeping genes and positive controls, used for normalization?**

To normalize the data, nCounter technology used:

1) Positive Controls (n=6). Probes that recognize synthetic mRNA targets included in the CodeSet at specified concentrations (targets do not require ligation). Positive controls used by the QC metrics in nSolver to confirm linear response to input amounts, and confirm that low input signal is above background.
2) Housekeeping genes: ACTB, B2M, GAPDH, RPL19, RPLP0.
   We have added the following explanation on p.6

3. **Let-7a, -7b and -7d were listed in Table 1 among a least variable miRNAs in thyroid gland, brain and as universal controls correspondently. But, miRNAs from let-7 family are well known tumor suppressors. Expression of these miRs is downregulated in tumors. How can it be possible that these miRNAs are the most stable?**

Analysis of the literature revealed that expression levels of members of the Let-7 family were found significantly different between tumor and normal tissue samples in several publications. The value of the difference varies both over different tissues and within individual tissues. The following ratios of concentrations were reported: Thyroid tumors (cancer sample versus normal): 0.61 (hsa-let-7d-5p) (Swierniak et al,2013); 1.21 (has-let-7f-1) (Pallante et al,2006); approximately 1.5 (has-let-7d) and approximately 1 (has-let-7g) (Braun et al,2010); approximately 3 (has-let-7a) (Zhou et al, 2017). Glioma (cancer sample versus normal): 1.69 (has-let-7b) (Zhang et al,2019), approximately 2 (has-let-7d and has-let-7a) (Yang et al). Bone marrow (cancer sample versus normal): (1.35-3.78) (Veryaskina et al,2020). We see that, in most of the cases, the difference between the expression levels was about 2 or less. In most of the studies, no correction for multiple comparisons was employed, which could lead to chance findings. Besides, generally, authors tend to mention more frequently miRNAs with significant differences than those for which comparisons yielded negative results. Therefore, it seems impossible to evaluate the number of studies where the differences for the Let-7 family miRNAs were not found significant.

On the other hand, only a small number of samples were used in our work. Therefore, the results are to be validated in succeeding studies.

It is also worth noting that let-7a, let-7b, and let-7d were not the most stable normalizers in our study.

4. **There is no confidence interval for p-values in the Table 2. Significant p-values in this table may be shown in bold or color.**

We might misunderstand the comment. As far as we know, confidence intervals for p-values were suggested only in some theoretical constructs and are not used in practice. Probably, the Reviewer suggested considering confidence intervals for the differences corresponding to the p-values. For Table 2, permutation tests were used, the differences being the sums of absolute differences of the corresponding ranks, which is described in the Statistical analysis section. As far as we know, there is no straightforward way to introduce confidence intervals for differences in permutations tests. Therefore, we did not present confidence intervals in Table 2.

The significant p-values are now shown in bold in Table 2.

5. **MiR-28-5p and miR-532-5p were chosen like the universal controls for all three tissues. But, among 10 least variable miRNAs, miR-28-5p is only ninth in thyroid gland and even was not listed in Table 1 for brain and bone marrow. The same story is for miR-532-5p. This miRNA is stable in brain tissues only. What is the possible reason for these discrepancies?**

For a miRNA, the variability in all tumors depends on within-group variability in each group and between-the-groups variability of the mean level of concentration of the miRNA. A miRNA may have low within-group variability in the groups, but high between-the-groups variability of the mean level of concentration; or vice versa. Therefore, the order of the miRNAs for all tumors may not correlate with the orders for individual tumors.

Reviewer #2

**The only thing that I would suggest is to correct the title into: Selection of reference genes for quantitative analysis of microRNA expression in three different types of cancers or even more specific in three types of cancers with thyroid, brain and bone marrow origin.**

According to the Reviewer’s suggestion, the title is corrected into: Selection of reference genes for quantitative analysis of microRNA expression in three different types of cancer.
